# Perceptions on Health Benefits of Guide Dog Ownership in an Austrian Population of Blind People with and without a Guide Dog

**DOI:** 10.3390/ani9070428

**Published:** 2019-07-07

**Authors:** Lisa Maria Glenk, Lucie Přibylová, Birgit Ursula Stetina, Sami Demirel, Karl Weissenbacher

**Affiliations:** 1 Comparative Medicine, The Interuniversity Messerli Research Institute, University of Veterinary Medicine Vienna, Medical University Vienna, University of Vienna, Vienna 1210, Austria; 2 Department of Clinical Psychology, Faculty of Psychology, Sigmund Freud University Vienna, Vienna 1020, Austria; 3 Coordination Center for Assistance Dogs, The Interuniversity Messerli Research Institute, University of Veterinary Medicine Vienna, Medical University Vienna, University of Vienna, Vienna 1210, Austria

**Keywords:** guide dog, dog ownership, quality of life, health, attitude, blind people

## Abstract

**Simple Summary:**

An emerging body of science has linked dog ownership with a better quality of life in their owners. However, there is limited information on the potential health benefits of guide dog ownership in blind people. This study sought to shed light on quality of life, annual medical costs, and attitudes towards the human–guide dog relationship in an Austrian population of 36 blind people with and without a guide dog. No significant differences in quality of life by means of a standardized questionnaire were found. Still, guide dog owners were more likely to regard a guide dog as a family member and to believe that guide dogs can increase their independency and, furthermore, have a positive effect on their health. Lower annual medical costs in guide dogs’ owners were reported on a non-significant level, as only few people provided the required information. These findings indicate that the attitude towards guide dog ownership varies between blind people with and without a guide dog. Further research into this topic is warranted.

**Abstract:**

Blindness has previously been associated with impaired quality of life (QOL). Guide dogs may not only support blind people in their independency, but also facilitate social relationships and overall health. This study sought to investigate whether blind people from Austria with a guide dog, when compared with blind people without a guide dog, differ in their QOL, annual medical costs, and attitudes towards the human–guide dog relationship. Participants (*n* = 36) filled out an online accessible questionnaire that consisted of the World Health Organization (WHO)QOL-BREF and additional self-designed questions. Guide dog ownership was not associated with a better QOL. However, yearly medical cost expenditures were descriptively lower in guide dog owners, who were also more likely to believe that guide dogs can increase their independency and exert positive effects on health. Moreover, guide dog owners more likely considered a guide dog as a family member than non-guide dog owners. Although within the framework of this study, owning a guide dog was not significantly associated with increased QOL, some differences between the groups regarding health beliefs, attitude towards the dog, and relationship with the dog were identified. Accounting for the emerging prevalence of visual impairment, further research into this topic is warranted.

## 1. Introduction

### 1.1. Quality of Life

The World Health Organization (WHO) has proposed that quality of life (QOL) is an individual perception of the position in life in the context of the culture and value systems in which a person lives and in relation to his or her goals, expectations, standards, and concerns. It is a broad-ranging concept affected in a complex way by the person’s physical health, psychological state, level of independence, social relationships, personal beliefs, and their relationship to prominent features of their environment [1] (p. 1). Borofsky and Rowan [2] understand QOL in its broadest sense as values held by an individual, a group, or an entire society. QOL assessment may be defined as the process of quantifying human values and incorporating them into important human decisions [2] (p. 93). Albrecht and Devlienger [3] described the “disability paradox”, where people with serious impairment assessed their QOL to be good or excellent, although they could appear to external observers as having a bad QOL. Therefore, a broader understanding of QOL, centered on the balance between body and mind, is feasible. Thus, it becomes apparent that QOL does not exclusively mean good health. Secondary, another complication with the described terminology is the relation between the terms “health” and “QOL” or “wellbeing”. The WHO defines health as “A state of complete physical, mental, and social well-being not merely the absence of disease or infirmity.” [1] (p. 1). Thus, it is the quality of life factor that is an important part of health as a whole. In other words, we cannot consider an individual as healthy if he or she is lacking the subjective feeling of wellbeing or contentment. Associated with the problem of finding the most appropriate definition of QOL, multiple research methods as well as new questionnaires may be considered [4]. In this respect, one of the most important methodological differences is the distinction between subjective and objective methods [5]. Objective methods focus, for example, on the measurement of income or the size of one’s flat [6], which are so-called social indicators used for evaluations of larger populations, by which different countries can be compared with specific regard to, for example, employment, access to health care, education level, or national security housing [7]. In contrast, subjective methods involve terms such as satisfaction [6] or economic, social, and psychological well-being [5]. Terms like objective and subjective measurement can be misleading. For example, somebody may have a tumour that has been measured by objective methods, but as long as the person does not know about his or her disease, the subjective QOL can be high while the objective QOL is low. Moreover, the objective methods are measured by external observers, whereas the subjective assessment is based on self-report [6], which is important because objective measurements do not always reflect the subjective ones [7]. Nowadays, mostly subjective methods are used, as they are more likely to capture the complexity of human life, which is missing in an objective assessment [8], or both methods are combined [9]. Questions should be relevant for the target group, sufficiently sensitive, and combined into discrete domains [8].

### 1.2. Visual Impairment

Although 79% of the Austrian population would evaluate their health status as “good“ or “very good“, 21% of the population perceive their health status as poor [10]. Most commonly mentioned problems include mobility, psychological and neurological problems, and visual impairment. Visual impairment is associated with an altered function of normal vision and there are various reasons that a person may be blind; however the most common eye diseases are glaucoma, diabetes retinopathy, tumors, and age-related macular degeneration [11]. Rubisch et al. [12] stated in 2017 that in Austria, 216,000 persons with visual disability (3% of the population between 15 and 60 years) were registered and women were more likely to be affected. A total of 53,000 (0.7%) of respondents had serious visual impairments and 2200 (0.03%) called themselves blind. Visually impaired people have to deal with permanent stress factors like dependence on others, helplessness, prejudices and insufficient social acceptance, or communicative problems on a daily basis [13]. Vuletić et al. [14] reported that partially sighted people have a better QOL and are significantly more satisfied with their close relationships than blind people. They are also more satisfied with achievements in life, health, standards of living, as well as future security and community connectedness. On the other hand, blind people reported higher safety feelings. Generally, both groups of visually impaired people were mostly satisfied with their close relationships and worried about their future security. It is important to mention that although partially sighted people reported a better QOL, both groups rated their QOL over 50% in all questions. A higher QOL was reported by blind people who were born blind or obtained blindness at a young age compared with people who acquired blindness later on. A higher QOL was also reported by people who joined a psychosocial rehabilitation group. Conversely, not visiting psychological rehabilitation was correlated with a lower feeling of security. These results are in agreement with results from Reboucas et al. [15], who investigated the QOL of visually impaired people in Brazil using the WHO-QOL-100 questionnaire. Similarly to the study conducted by Vuletić et al. [14], the participants assessed their QOL as good (68.75%). In a certain contrast to the above-mentioned studies, Van Nispen et al. [16] reported that visually impaired people suffer from a higher risk of depression with increasing vision loss and proposed an association between social network size and depression. In general, having a partner was found to be protective against depression. Similarly, Langelaan et al. [17] associated visual loss with depression and anxiety in patients who lost vision before the age of 12, or whose visual impairment began before this age. Regardless of the degree of visual impairment, Kamelska and Mazurek [4] found that physical activity can significantly improve the QOL of visually impaired people. They consider physical activity as a possibility to explore own personality traits and develop creativity. Moreover, finding motivation and the possibility to overcome difficulties associated with visual impairment also shapes social integration if training partners are available.

### 1.3. Dog Ownership and Health Benefits

According to Kurdek [18], adult dog owners turn to their dogs rather than to close friends or relatives in times of emotional distress. A total of 79% of pet owners stated that their pet helps them to get through the difficult times, as the majority of owners considered their pets to be a part of the family [19]. Pet ownership has been repeatedly linked to increased health by means of fewer doctor visits and less use of medication and, as a result, pets could possibly save national health expenditures [19,20,21]. Potential health benefits of assistance dogs are often overlooked by medical professionals, as the health management is primarily focused on pharmacological and surgical treatments. The most common mobile aid associated with blind people is the white cane, and although the cane is a very practical mobile aid, an overwhelming majority of guide dog owners preferred guide dogs over the cane [13,22,23]. In addition, although nowadays many technology devices for mobility like global positioning system (GPS) or talking sight exist, guide dog owners prefer guide dogs even over those modern mobility aids [23]. They perceive guide dogs as a safer and faster mobility aid, especially in unknown environments [24]. As Spence [25] suggests, service dogs offer even more advantages than pet dogs. It is necessary to highlight the fact that pet dogs do not have public access rights. Moreover, service dog owners reported a higher QOL when compared with pet dog owners with impaired mobility. Refson et al. [26] studied the health status of guide dog owners and non-dog owners in Scotland. The study results propose that guide dog owners had a significantly better health in comparison with non-dog owners. Lane et al. [27] suggested enhanced self-perceived health in the service dog group, while a majority of the participants of the study reported to be more relaxed since the dog was present and they also worried less about their health. They explained the health improvement as follows: “Reports from many of our subjects suggest that an enhanced sense of physical and psychological health may be associated with the role of their dog as a means of social integration, a close affectionate companion and a source of support and comfort” [27] (p. 56). The presence of guide dogs implicitly leads to increased physical activity. Visually impaired people walk longer distances with a dog than they would if only using a cane [23]. In contrast to this finding, in another study, only seven people out of 80 respondents reported that the guide dog promoted better health and increased fitness [13]. In previous studies, the most frequently discussed topics are increased independence and the possibility to find new social contacts thanks to the presence of a guide dog, rather than a direct impact on improved health or economic cost issues. This is except for one U.S. publication, which dealt directly with the economic costs and financial benefits of guide dogs, estimating the total costs per one guide dog over one working year to be about 2379 USD. These results were calculated after the summation of all costs for a guide dog, like costs including acquisition and training of the dog as well as annual maintenance costs over the dog’s life. People who owned a guide dog needed less formal and informal care. Therefore, the total discount of formal (16,324 USD) and informal (5244 USD) care was 21,568 USD per year [28]. Economic benefits of service dogs were also presented in an earlier study by Fairman and Huebner [29], who reported an average reduction of two hours of human paid assistance per week and six hours of unpaid assistance per week, which is equivalent to 600 USD per year. Although the studies presented above reported many positive facts about guide dog ownership, not all visually impaired people have or want a guide dog. One study from the U.K. pointed out the discrepancy between the prevalence of visual impairment and actual guide dog ownership. Several reasons may account for the fact that not all visually impaired or blind people have a guide dog. The most common are informational, psychological, social, and environmental barriers [22]. Lane et al. [27] investigated the most motivational factors in applying for a dog and 70% of participants of the study responded that they hoped to be more independent, 35% responded that they wanted companionship, and 23% hoped to be able to socialize more. Visually impaired people who do not own a guide dog may not be able to imagine how much of a friend the guide dog may be, nor his or her ability to facilitate contact with other people [13]. Consequently, the most common incentive for applying for a guide dog is increased independence and improved mobility, not the companionship [26]. As a result, the guide dog usually holds a positive surprise for the future guide dog owner [13]. The relationship between the guide dog and the owner seems to play a role in the perception of the positive effects of the dog. Owners who possessed similar personality traits as their guide dogs reported more satisfaction with their dogs [30,31]. Moreover, guide dog owners who acquired a guide dog of their own free will reported greater satisfaction with the dog’s work, as well as with the mutual relationship. In addition, 93% of respondents had a good relationship with the dog and they even valued the dog’s importance to be similar to that of family members [27]. Similarly, Wong [23] reported that all respondents except one considered their guide dog as a companion and friend. Previous studies have also suggested that the presence of a dog can facilitate social contacts by increasing the social attractiveness of his or her handler [32,33,34].

### 1.4. Guide Dogs in Austria

According to the Austrian law, a guide dog may support and assist a blind person or visually-impaired person, and thereby increase his or her mobility. To award an official certificate, all guide dogs have to be examined by the independent Coordinating Authority, located at the Messerli Research Institue, assigned by the Social Ministry. After the exam has been successfully passed, the guide dog can be written into the owner’s passport for a person with disability. At that time, the guide dog is officially accepted by the Austrian Republic as an assistance animal according to § 39a BBG [35,36,37]. The guide dog obtains a number and is listed in the guide dog database [36]. Since 1 January 2015, when the law about guide dogs came into force, the Coordinating Authority launched the guide dog assessment service. Guide dog statistics reveal that in the year 2015, 21 guide dogs who had been already working as a guide dog at that time were tested. In addition, 12 guide dogs passed the exam in the year 2015. In 2016, another 13 guide dogs passed the exam; in 2017, 11 guide dogs successfully completed the examination; and in 2018, 14 guide dogs passed successfully. It is estimated that in Austria, there are about 78 working guide dogs that passed the official exam and about 30 guide dogs that worked already before the official exam was launched and thus did not take the exam. In total, approximately 108 guide dogs are working in Austria [38], of which 72.2% are officially certified. Financial support is available from the Social Ministry, however, actual costs for a guide dog range between 34,000–40,000 EUR [39]. Thus, there is a considerable financial burden associated with the adoption of a guide dog.

### 1.5. Study Aims

Although there is some preliminary evidence that dog ownership may increase the QOL in guide dog owners, no previous study on this topic was done with an Austrian cohort. Moreover, scientific evidence on the attitude towards the relationship with a guide dog and whether a guide dog may effectively support the independence and perceived health of a blind person is also lacking.

Thus, the primary aim of this study was to investigate whether blind people having a guide dog have a better QOL compared with blind people not having a guide dog. Moreover, we sought to investigate whether blind people who have a guide dog are healthier and thus have lower medical costs per year. Finally, we aimed to shed light on the attitude towards the human–guide dog relationship, that is, how respondents of both groups rate the human–guide dog relationship and believe in its positive effects.

## 2. Materials and Methods

### 2.1. Study Participants

For the purpose of this study, an accessible online questionnaire was used, advised by a blind volunteer who was involved in the planning and data collection phase. Blind people with and without a guide dog were invited via distribution of an invitation letter by official organizations according to the General Data Protection Regulation (GDPR) law.

Online questionnaires are fully anonym and barrier-free for everybody who has access to the Screen Reader software, an assistive technology essential to people with visual impairment. Furthermore, while the voice of the researcher may influence the respondent, the Screen Reader voice is artificial and emotionless. This is another huge advantage of the method, as blind people could be more sensitive on auditory cues. Nowadays, diverse companies such as Apple, Google, or Microsoft have included the Screen Reader in their system. Some Screen Reader software programs are open source and freely available to download [40,41]. During the recruitment phase (2 September 2018–30 November 2018), potential participants of the first group were contacted via the Coordination center for assistance dogs in Austria (https://www.vetmeduni.ac.at/de/assistenzhunde/), because thanks to the mandatory exam of all Austrian assistance dogs, all service dog owners are registered in this database. The second group was contacted via the Association for blind and visually impaired people in Austria (http://www.blindenverband.at/). All potential participants obtained an invitation email via the above-mentioned organizations. The invitation email contained a link that led the participants to a website with the questionnaire.

Blind adults between 18 and 65 years old were eligible for participation. An age limit of 65 was set because of a higher risk of co-morbidities and overall mortality. According to Austrian Statistics, 16% of the Austrian population over the age of 65 are likely to have problems with at least one basic activity like eating or taking a shower [10]. All participants had to live in Austria because it would not be feasible to compare the QOL across different countries according to overall life standard and culture. The inclusion criteria for the group with a guide dog was having a guide dog and for the group without a guide dog, the inclusion criteria not having a guide dog at the moment or within the past three years. Two groups of blind participants completed the accessible online questionnaire. The first group consisted of blind people with a guide dog (*n* = 18) and the second group of blind people without a guide dog (*n* = 18).

### 2.2. Instruments

For the purpose of this study, two versions of the questionnaire were used. The first part contained identical questionnaires for both groups, based on the German version of the WHOQOL-BREF (World Health Organization, Quality of life questionnaire a shorter version of the original instrument WHOQOL-100 questionnaire). The second part was a questionnaire designed for the specific purpose of this study. Questionnaires were accessible via an online link. For this purpose, we used (https://www.soscisurvey.de/), which is available freely to students to publish their questionnaires. Soscisurvey is also compatible with the Screen reader modus, which enables visually impaired people to use a computer.

#### 2.2.1. WHOQOL-BREF

The WHOQOL-BREF is a brief version of the WHOQOL-100 (World Health Organization, Quality of life questionnaire). It was developed for use in larger studies and for cases in which the use of a longer version is not practicable [1]. On average, the WHOQOL-BREF should be completed in 5–10 min [42]. As this study is focused on blind people, the longer version WHOQOL-100 could be to exhaustive; therefore, it was decided to use the shorter WHOQOL-BREF. The WHOQOL-BREF has been translated into over 30 languages to allow for comparative and cross-cultural studies [43]. It is a useful instrument to assess individual perception of QOL. The questionnaire contains 24 items, which are assessed within four domains: physical (seven items), psychological (six items), social relationships (three items), and environment (eight items), plus two items scored separately. The WHOQOL-BREF German version was requested for the purpose of this study from the WHO organization, and its content was changed to a barrier-free version made suitable for blind people with the help of a blind volunteer.

#### 2.2.2. Self-Designed Questions

The second part of the questionnaire was specifically designed for the purpose of this study. Both groups obtained a tailored version of the questionnaire. The group with a guide dog was asked how the presence of the dog influenced their daily life, meaning their sufficiency, their access to new social contacts, and their perceived health. The next questions were associated with the dog, whether participants consider it demanding to own a guide dog, whether they think that it is a demanding job for the dog, and about their general relationship with the dog. Prior to access to the questionnaire, a filter system screened for guide dog ownership. If the participant answered yes (having a guide dog), They were automatically directed to the questions for the group with a guide dog. If the answer was no (not having a guide dog), the participant was automatically directed to the questions for the group without a guide dog. Participants of the second group without a guide dog were asked basically the same questions as the first group, just in a hypothetical way as if they imagined they had a guide dog. Agreement on those questions was rated on a 10-point scale—1 for “none” and 10 for “high”. In addition, participants were asked if they suffer from any chronic diseases, if they consume any addictive substances, and about their medical cost in the year 2016 according to medical insurance. A chronic disease was defined as a continuing condition over a six-month period. Among the most common chronic diseases in Austria are back pain, allergies, high blood pressure, chronic cervical spine pain, arthrosis, depression, headache, diabetes, asthma and chronic bronchitis, and heart attack [10]. For the purpose of this study, all types of pain were scored in one item except for headache, which was assessed separately, and questions about cancer and anxiety were added.

### 2.3. Ethical Consideration

The study protocol was approved by the Medical University of Vienna Human Participants Ethics Committee, reference number 1247/2018 (submitted by Dr. Lisa Maria Glenk). No individual-related questions (e.g., birth date or name of the participant) were included in the questionnaire. At the end of the questionnaire, participants had to confirm that they understood that their data will be anonymously analyzed solely for purpose of this study.

### 2.4. Statistics

All analyses were performed using IBM SPSS (version 24.0; IBM Corp., Armonk, NY, USA). Differences between the two groups in frequency distribution were analyzed using chi-square tests. For metric scaled variables such as the domain scores of the WHOQOL-BREF, the two groups were compared using t-tests for independent samples or the nonparametric Mann–Whitney-test (U-test), if the assumption of normal distribution, which was tested using Kolmogorov–Smirnov-test, was not met, or in the case of ranked data. For all analyses, a *p*-value of 5% (*p* > 0. 05) was considered significant. On the basis of power analysis and expected responses, a minimum sample of 16 participants was required for each group (standard deviation: 2; group 1 mean: 7; group 2 mean: 5; power (1−β): 0.8; type error rate α: 0.05).

## 3. Results

### 3.1. Sociodemografic Data

In total, 36 participants finished the questionnaire. According to intern SoSci statistics, which keep in record how many times the online questionnaire has been accessed as well as successful and unsuccessful attempts of completion, 51 questionnaires were not successfully finished. In total, the online questionnaire was opened 475 times. The socio-demographic data of the participants are presented in Table 1. The data are sorted by the tested group: group without a guide dog (NGD) and group with a guide dog (GD). The size of the groups was equal, with 18 participants in each group. In total, more females attended the study, as 12 females and only 6 men were included in the GD group. In the NGD group, gender was equally balanced. The mean age in GD group was 42.1 years and 48.9 years in the NGD. The majority of the respondents was either single, divorced, or widowed. In both groups, 15 respondents stated that they were currently not ill and three respondents stated that were currently ill.

### 3.2. Quality of Life

The results regarding QOL obtained from the WHOQOL-BREF, which refers to a self-assessment of QOL, are shown in Table 2. No significant differences between groups were observed in any of the domains (physical, psychological, social relationships, environment). Respondents self-assessed their QOL in general as good. In all domains, they assessed their QOL over 50%. According to the ordinary scale defined by WHO, 50% can be defined as neutral (neither good nor not good). According to the results of this study, the environmental domain reached the highest score of all domains in both groups: 70.7% within GD the group and 70.8% within the NGD group. High scores were also found in the physical domain (65.7% within the GD group and 71% within the NGD group). The social relationships domain reached 68.1% in the GD group and 66.7% in the NGD group. The psychological domain was the only one that did not reach more than 60%. The NGD group assessed the psychological QOL with 55.5% and the GD group with 52.3%.

### 3.3. Health Status and Consumption of Addictive Substances

Respondents were asked whether they suffered from chronic illness like chronic pain, asthma, allergy, diabetes, depression, high blood pressure, chronic headache, arthrosis, chronic bronchitis, cancer, stroke, anxiety, or any other chronic diseases. There was no significant difference between both groups regarding suffering from chronic illnesses. Nevertheless, it could be observed that GD group suffered non significantly more under depression, high blood pressure, and anxiety. A totoal of 38.9% of respondents of the GD group reported that they suffer from depression, whereas only 16.7% of the respondents of the NGD group reported this. A total of 27.8% of the GD group reported that they had increased blood pressure, while in the NGD group, 16.7% of subjects reported increased blood pressure. Increased anxiety was reported by 38.9% of respondents of the GD group in contrast to the NGD group, where 22.2% respondents reported increased anxiety. All other responses regarding chronic pain, asthma, allergy, diabetes, chronic headache, arthrosis, chronic bronchitis, cancer, stroke, and other chronic disease were not significantly different between the groups. Regular usage of medication was reported by 72.2% of respondents in the GD group and by 68.8% in the NGD group. This study showed no significant difference regarding the consumption of addictive substances between both groups. Respondents were asked if they consume any of the following addictive substances: alcohol, nicotine, caffeine, cannabis, stimulants drugs, sedative drugs, or any other drugs. Nevertheless respondents of the GD group reported non-significantly higher consumption of nicotine, cannabis, stimulants drugs, and sedative drugs. According to the results, 38.9% of the respondents from the GD group consumed nicotine in comparison with 16.7% in the NGD group. A total of 22.2% of the GD group consumed cannabis, whereas 11.1% of the NGD group respondents did. Usage of stimulants and sedatives drugs was in general very low; 5.6% of the GD group respondents consumed stimulant drugs, whereas no respondent of the NGD group reported usage of stimulants. Consume of sedative drugs was reported by 11.1% of the GD group respondents and by 5.6% of the NGD respondents.

### 3.4. Medical Insurance Costs

Respondents were asked about their medical costs by means of medical insurance expenditures from the year 2016. This question was not obligatory in terms of inclusion criteria to take the questionnaire as valid. Only 10 participants provided information about their medical insurance costs, three participants from the GD group and seven from the NGD group. With that limited information, no statistical analysis was possible, but on a descriptive level, higher costs emerged in the NGD group. The mean medical costs in the GD group were 492.67 (± 467.249 SD) EUR per month, whereas in the NGD group, it was 2445.98 (± 3146.154 SD) EUR.

### 3.5. Owner–Guide Dog Relationship

Respondents self-reported their relationship with their guide dog or, in the case of the NGD group, on a hypothetical basis. As depicted in Table 3, statistically significant differences were observed between both groups in questions (Q)1, 3, and 6. The most striking difference in the responses between the groups was seen in Q1 (To what extent did the presence of the dog increase your independence?), where the mean score in the GD group was 8.67, whereas in the NGD group, it was significantly lower with 3.83 (*p* < 0.001). In Q3 (To what extent did the presence of the dog improve your health?) and Q6 (To what extent do you consider the guide dog as a family member?), there were also significant differences between both groups. The majority of the GD group respondents reported that their health was improved since they had a guide dog, whereas the majority of NGD group respondents did not believe that the presence of a guide dog could improve their health. According to Q6, all respondents of the GD group considered their guide dog as a family member and scored lowest with number 7 on the ordinary scale, while NGD group respondents scored also with number 1, although the general mean of the NGD group was also relatively high (8.33 in contrast to the mean of 9.83 of the GD group). Except Q5 (To what extent do you think that the service of the guide dog negatively influences its QOL?), all relationship-related questions were scored higher by the GD group than by the NGD group. Although there was no significant difference between both groups with regard to Q2, a tendency was found that respondents of the GD group responded more often that a guide dog facilitates finding new social contacts in comparison with the NGD group.

## 4. Discussion

Accounting for the global prevalence of visual impairment, research into the feasibility of guide dog provision and funding is needed. The results of this study suggest that blind people with a guide dog do not have a better overall QOL than those without a guide dog by means of the WHOQOL-BREF. Again, no significant differences were observed when any of the isolated domains (physical, psychological, social relationship, and environment) were compared between the groups. The results of this study are in agreement with the study from the authors of [15], who investigated the QOL of visually impaired people in Brazil using the WHOQOL-100 questionnaire. The results of their study reported a similarly high scored QOL in visually impaired people, like in this study. Brazilian visually impaired respondents rated their QOL as 68.75%. It is interesting to note that respondents of the Brazilian study scored higher in the psychological and social relationships domain, but lower in the physical and environmental domain compared with this study on Austrians. A big difference was observed in the environmental domain, in which Brazilian respondents scored with 48.48%, whereas Austrians with 70.7% (GD group) and 70.8% (NGD group). This difference may be attributed to the different public infrastructure in these countries. In Brazil, the public infrastructure is relatively poor [44], and it can be difficult for blind people to orientate themselves in the traffic, whereas in Austria, a wide range of accessibility by road, air, and rail is available [45]. In addition, Vuletić et al. [14] suggested that the QOL score of visually impaired people in Croatia was rated as good (68.75%); this result is within the normative range for the global population of 60% to 80%. The high rated QOL in visually impaired people could also be explained by the disability paradox. Despite severe impairment, people with disability can achieve a high QOL, when they are able to center the balance between their body and mind [3]. However, it is suggested that visually impaired people may benefit from a wide array of support, for instance, disabled individuals who visited psychological rehabilitation reported a higher QOL [15]. On the contrary, the results of this study regarding the general QOL, which was rated as good in both groups, are in contrast to several previous publications. For instance Langelaan et al. [17] reported a lower QOL in visually impaired people. Our study results are also in disagreement with Hall et al. [46], who found that people owning a hearing or physical service dog rated their QOL significantly higher. Enhanced psychological and physical wellbeing in guide dog owners, which was also reported by Refson et al. [26] and Whitmarsh [22], could not be confirmed in the present analysis. A plausible reason for not having achieved any statistical differences in QOL between the GD and the NGD group in this study, besides the relatively small sample size, is that possibly only active individuals with a fulfilled life were willing to participate in such a study. With the presumption that active visually impaired people have eventful lives and sufficient social contacts, a better QOL is likely, and thus no difference between the groups was found. It would be worthwhile to explore this assumption in future studies by analyzing the study respondents’ overall life satisfaction including social networks and job efficiency. In addition, owning a pet other than a guide dog could have shaped the responses, so that pet ownership should definitely be controlled for in continuative research. Another explanation may be associated with the initial QOL scores, in that values above a certain threshold are unlikely to rise even in response to a positive stimulus such as the presence of a dog. Still, there seems to be a discrepancy between the objective QOL score and the subjective interpretation as guide dog owners, if they are asked whether their QOL was improved since the dog’s presence, answer yes. Similarly, Wong [23] reported that guide dog owners believe that their guide dog changed their life positively. Maybe, when the respondents with guide dogs assessed their QOL with a less direct question about QOL using the WHOQOL-BREF questionnaire, they scored their QOL similar to non-guide dog owners although believing that the dog raised their QOL. Likewise, the results of this study indicate that guide dog owners believe that their health was improved since the presence of their dog (this refers to Q3, which will be discussed more in detail later on), but when assessing the chronic illnesses, no difference was observed.

Unfortunately, the question on yearly medical costs could not be answered on a statistically significant level, as many respondents failed to provide their medical insurance fees. Thus, GD group respondents had lower medical costs only by means of descriptive data analysis. Information about medical costs was not obligatory in terms of an inclusion criterion to complete the questionnaire, and only 10 participants in total provided this information. Insurance companies provide each of their clients with the yearly medical costs statement per post and it is possible to obtain this information online, so it was assumed that study respondents can easily access their respective costs and include this information. However, it turned out that the majority of participants failed to provide this information. In future studies, it should be taken into consideration that access to the medical yearly costs statement can be facilitated so that the participants are not burdened with obtaining this information. From another perspective, personal medical costs may be regarded as too sensitive and, as a result, participants possibly were not willing to provide this information. If this was the case, then in future studies, it would be necessary to explain more concisely that this information is handled as anonymously as the entire questionnaire to enhance the trust of study volunteers. In the absence of statistical significance because of the few respondents, higher numbers of yearly medical costs were given in the NGD group. This is in line with earlier findings by Jackson et al. [47] and Refson et al. [26], who reported better health in guide dog owners. Serpell [48] compared in a 10-month prospective study the general health and psychological wellbeing before and after acquiring a dog and concluded that dog owners improved in health and wellbeing. Controversially to these findings, Steffens and Bergler [13] suggested no health promotion in guide dog owners. There are no studies to the best of our knowledge that calculated the general economic benefits of guide dogs regarding health savings, and savings in formal and informal assistance care. One study calculated the economic benefit of guide dogs regarding formal and informal care, and reported those savings of about 2379 USD per working year of a guide dog [28]. Nevertheless, such calculations are conflicting as actual health expenditures were not considered. Although at first look, the guide dog costs are high, possible savings in national health expenditures could be interesting for the insurance companies and their willingness to financially support guide dogs or other assistance dogs as valid medical adjuvant. To acquire more data, future investigations are necessary.

In this study, no significant differences between the groups were observed regarding the suffering from chronic illnesses, as well as regarding the injecting of addictive substances. Nevertheless, descriptive data analysis revealed higher depression, and high blood pressure and anxiety scores in the GD group. Van Nispen et al. [16] reported a higher risk of depression in visually impaired people in general. Our findings are in agreement with the results of Parslow et al. [49], who concluded that pet ownership had no health benefits and was associated with symptoms of depression. In addition, Parslow and Jorm [50] found no differences in systolic blood pressure between the groups of pet owners and non-pet owners, and pet owners were associated with higher diastolic blood pressure. Milan [51] concluded that there is no association between levels of depression and mobility dog ownership. However, accounting for the positive effects of dog companionship on human health [18,19,20,21], it is also possible that people with higher levels of depression, anxiety, or blood pressure are more likely to apply for a guide dog in the hopes of alleviating those conditions. Only descriptive data analysis revealed higher injecting of addictive substances like smoking behavior, and consumption of cannabis, stimulants, and sedative drugs in the GD group. Increased smoking behavior was also observed in a previous study. According to Parslow and Jorm [50], pet owners reported increased smoking behavior. Nevertheless, usage of addictive substances is generally not well investigated in this scientific field, and should be further explored.

This study aimed to further compare blind people with and without a guide dog with regard to differences in their attitude regarding the relationship towards the guide dog. It emerged that actual guide dog owners are more likely to believe in the positive effects of this relationship. The results suggest statistically significant differences between both groups in some of the questions of the self-designed instrument, (Q1—To what extent did the presence of the dog increase your independence? Q3—To what extent did the presence of the dog improve your health? Q6—To what extent do you consider the guide dog as a family member?). The biggest difference in the responses between the GD and NGD group was found in Q1, with *p* < 0. 001, where the GD group scored significant higher compared with the NGD group. Although it was expected that guide dog owners will score higher in this question, the magnitude of the effect was surprising, as increased independence due to the presence of a guide dog is highly discussed in previous studies, as well as among the non-scientific population. Refson et al. [26] reported higher independence in guide dog owners compered with visually impaired non-guide owners. Similar results were presented by Hall [46], with the difference that the study assessed assistance dogs in general, not only guide dogs. An increased level of independence was also found by Steffens and Bergler [13]. Moreover, Lane et al. [27] noted that 70% of people who applied for a guide dog did so because they hoped for greater independence. Similarly, Refson et al. [26] suggested that the hope for independence is the most common motivation for applying for a guide dog, and not companionship or other possible benefits. The benefit of independence was reported especially in male guide dog users [22]. There was also a significant difference regarding the results of the owner–guide dog relationship and the extent that respondents believed that the presence of the dog improved their health. The GD group scored significantly higher compared with the NGD group. Again, these results confirm those of a previous study. According to Lane et al. [27], enhanced self-perceived health and less worries about health were found in service dog owners. Accordingly, in future studies, it would be interesting to compare the self-perceived health with objective assessed health more in detail. Another significant result relates to the attitude towards the dog, whether or not respondents considered a guide dog as a family member. Again, the GD group scored significantly higher compared with the NGD group. These findings are in agreement with the results of previous studies. Similarly to this study, Lane et al. [27] stated that 93% of the participants rated the dog’s importance similar to that of family members. Future guide dog owners (those who apply for a guide dog) usually do not expect that a guide dog will become a family member [13,26]. Moreover, the association between health benefits and the relationship between the owner and dog does play a role [19,30,31], meaning those with a better relationship with their dog benefit more from it. In addition, the beneficial effect was intensified when it was the owner’s own idea to acquire a guide dog [27].

In Q2 (To what extent did the dog facilitate to find new social contacts?), a non-significant trend with *p* < 0. 081 was observed. GD group respondents stated more often that a guide dog facilitates finding new social contacts compared with the NGD group. This finding may be because of the lack of real experience with a guide dog in the NGD group, as previous studies have shown that canine companions can stimulate prosocial behavior of strangers, and thereby raise the social attractiveness of the animal handlers [32,33,34]. Guide dogs should not interact with people in the public to keep levels of distraction low. However, strangers may still be attracted by the dog, and thus want to approach or pat it, and thereby initiate contact with the animal handler [30].

Regarding Q4 (To what extent do you think that the service of the guide dog is demanding?), Q5 (To what extent do you think,that the service of the guide dog negatively influence its QOL?), and Q7 (To what extent do you consider the guide dog as a medical adjuvant/mobile aid?), no significant differences were found. Regarding Q4, the GD group had lower ratings than the NGD group. Although this result was not significant, it is interesting that guide dog owners were unlikely to regard the guide dog service as demanding. According to Craigon et al. [30], guide dog owners agreed that their dogs enjoyed their work. Guide dog owners may be more likely to think that if their guide dogs want to work, their service is not demanding. Although blind people cannot directly observe their dog’s behavior, guide dog owners register emotional states relevant to dog well-being such as tension or calmness, as suggested by Craigon et al. [30]. However, accounting for animal welfare, research on how blind people discriminate canine stress behaviors would be desirable. Although Q5 was rated higher on a descriptive level in the NGD group, in which participants believed more often that the service of the guide dog can negatively influence its QOL, this question was in general low-rated. In contrast, Q7 was rated very high in both groups, which could be indicative for an attitude towards an instrumentalization of the dog. Nevertheless, a guide dog can be seen as both a family member and a mobile aid. In general, the topic of guide dogs QOL was not the focus of this study, but nevertheless should be discussed in future studies. In summary, although visual impairment is not generally linked to a poor QOL [17], there are several aspects, including physical activity, psychological support, social network size, the time point of vision loss, and whether he or she is partially or full blind, that can improve the QOL [4].

### Limitations of the Study

A limitation of the study is the relatively small sample size, although the minimum sample size determined by power analysis was met. Participants from the GD group were recruited via an invitation email via the Coordination center for assistance dogs in Austria, whereas participants from the NGD group were contacted via the Association for blind and visually impaired people in Austria. The Coordination center for assistance dogs in Austria sent reminder emails to all (78) potential participants. Nevertheless, only 18 guide dog owners completed the questionnaire. The Association for blind and visually impaired people in Austria sent invitation emails only. As the GDPR came into force before the invitation emails were sent, some of the coordinators of the federal states of Association for blind and visually impaired people in Austria were no longer willing to forward an invitation email. This may have resulted in a low participation rate. Finally, the head of the Association for blind and visually impaired people in Austria shared the invitation email with all members of the Association for blind and visually impaired people in Austria. Nevertheless, there were also only 18 participants in the NGD group. In the case of the GD group, however, 18 subjects represent a sizeable sample of the blind population with a guide dog. As previously mentioned, there are around 78 officially tested guide dogs in Austria [38], but in the NGD group, it is questionable whether the results can be generalized to the blind population at large. In addition, 51 participants did not finish the questionnaire. This may be attributable to the length of the questionnaire, which some participants may have considered as too time-demanding. Also, the questionnaire contained sensitive questions and, although these were anonymized, some participants may have not been willing to respond to them. Moreover, invitation via an official association could have influenced the selection of participants.

As a research method, an online questionnaire was used. Another possibility would be a telephone interview or a personal interview. The advantage of using telephone or personal interview is that participants who do not use a computer can be easily reached. However, nowadays, computer usage among the blind community is very common. According to the Screen Reader Survey, the majority of people with impairment who use the Screen Reader are in fact people with visual impairment [52]. Modern technologies are thus extremely helpful for blind people to be independent and use the Screen Reader rather than depend on the help of other people. Therefore, the questionnaire of this study was designed according to the Web Content Accessibility Guidelines (WCAG) guidelines for barrier-free access [53]. Moreover, the disadvantage of a telephone interview is that they are more time-demanding and, in interaction with the interviewer, respondents may be more shy with regard to their privacy. Another benefit of the questionnaire is that respondents do not get influenced by the voice of the experimenter, which cannot be standardized and may contain emotional cues.

The self-administered questionnaire on the human–guide dog relationship was an innovative approach, in which actual guide dog owners’ attitudes and experiences were compared to hypothetical answers of non-dog owners. While the significant findings are interesting and certainly provide a starting point for continuative research, there is no evidence whether hypothetical pet ownership can be regarded as a valid construct to allow for comparison. Limitations of this study include also the fact that this study was not longitudinal. It would be more effective to assess the QOL of future dog owners and compare these results later on, when they live with the dog for a longer period of time. In the future, longitudinal studies with larger sample sizes should be conducted. Financial benefits or other benefits could be used to motivate more people to participate in such studies. It would also be beneficial if insurance companies would be involved to provide the medical information to the participants more easily.

## 5. Conclusions

Using the standardized questionnaire for the assessment of QOL (WHOQOL-BREF) and a self-designed questionnaire for assessing the attitude and relationship towards the guide dog, this study documented the QOL of blind people with and without a guide dog, their health status, as well as the attitude and relationship towards guide dogs. Owning a guide dog was not significantly associated with a better QOL. Although, unfortunately, not all participants provided the information about yearly medical costs, guide dog owners reported lower medical costs. When assessing the participant’s attitude regarding the relationship towards the guide dog and his or her belief in the positive effects of this relationship, some statistical differences between the groups were observed. The findings from this study propose that compared to non-dog owners, blind people with a guide dog in Austria are more likely to believe that the dog can increase their health and independence and, to some extent, facilitate social contacts. However, there is further research needed to investigate whether there are causal effects. Future studies with larger sample sizes would be beneficial for visually impaired people and their families, insurance companies, and governments to achieve a better understanding of guide ownership benefits, potential savings, and expenditures regarding guide dogs.

## Figures and Tables

**Table 1:** Socio-demographic data of study participants with (GD) and without a guide dog (NGD) in Austria.

Participants (*n* = 36)	GD	NGD
Gender		
Male	6	9
Femal	12	9
Age		
18–30	1	4
30–40	4	4
40–50	2	5
50+	10	4
Mean Age	42.1	48.9
Marital Status		
Single	6	8
Married	3	5
Living with partner	2	4
Divorced	4	1
Widowed	3	0
Highest Received Education		
Secondary school	4	2
General secondary school	5	2
Advanced technical college entrance qualification	1	0
Maturity	2	8
College of higher education	2	2
University diploma	4	3
Doctorate/PhD	0	1
Currently Ill Status		
Yes	3	3
No	15	15

**Table 2:** Results of World Health Organization Quality of Life (WHOQOL)-BREF. Comparison of four domains between study participants with (GD) and without a guide dog (NGD) in Austria.

WHOQOL-BREF Domain	Group	Mean	SD	*p*
Physical	GD	65.7	21.0	0.461
	NGD	71.0	22.0	
Psychological	GD	52.3	16.2	0.600
	NGD	55.3	17.9	
Social relationships	GD	68.1	19.0	0.835
	NGD	66.7	20.6	
Environmental	GD	70.7	19.9	0.977
	NGD	70.8	16.3	

**Table 3:** The results of owner–guide dog relationship/hypothetical relationship in study participants with (GD) and without a guide dog (NGD) in Austria. * denotes statistical significance.

Self-Designed Questions	Group	Mean	SD	Median	Min.	Max.	*p*
1. To what extent did the presence of the dog increase your independence?	GD	8.67	1.715	9.5	4	10	<0.001 *
	NGD	3.83	3.468	2	1	10	
2. To what extent did the dog facilitate to find new social contacts?	GD	8.06	2.209	8.5	2	10	0.081
	NGD	5.67	3.819	6	1	10	
3. To what extent did the presence of the dog improve your health?	GD	7.72	2.081	8	4	10	0.021 *
	NDG	4.78	3.719	3.5	1	10	
4. To what extent do you think that the service of the guide dog is demanding?	GD	6.22	2.463	5.5	3	10	0.513
	NGD	6.56	2.684	7	1	10	
5. To what extent do you think that the service of the guide dog negatively influences its QOL?	GD	3.28	2.469	2.5	1	9	0.203
	NGD	4.44	2.895	4.5	1	10	
6. To what extent do you consider the guide dog as a family member?	GD	9.83	0.707	10	7	10	0.005 *
	NGD	8.33	2.828	9.5	1	10	
7. To what extent do you consider the guide dog as a medical adjuvant/mobile aid?	GD	7.17	3.569	9.5	1	10	0.715
	NGD	6.89	3.142	7.5	2	10	
